# Importance of Increased Arterial Resistance in Risk Prediction in Patients with Cardiovascular Risk Factors and Degenerative Aortic Stenosis

**DOI:** 10.3390/jcm10102109

**Published:** 2021-05-13

**Authors:** Jakub Baran, Paweł Kleczyński, Łukasz Niewiara, Jakub Podolec, Rafał Badacz, Andrzej Gackowski, Piotr Pieniążek, Jacek Legutko, Krzysztof Żmudka, Tadeusz Przewłocki, Anna Kabłak-Ziembicka

**Affiliations:** 1 Department of Interventional Cardiology, Institute of Cardiology, Jagiellonian University Medical College, John Paul II Hospital, 31-202 Krakow, Poland; jakub_baran@yahoo.pl (J.B.); kleczu@interia.pl (P.K.); lniewiara@gmail.com (Ł.N.); jjpodolec@gmail.com (J.P.); rbadacz@gmail.com (R.B.); kardio@kki.krakow.pl (P.P.); jacek.legutko@uj.edu.pl (J.L.); zmudka@icloud.com (K.Ż.); tadeuszprzewlocki@op.pl (T.P.); 2 Department of Emergency Medicine, Faculty of Health Sciences, Jagiellonian University Medical College, 31-126 Krakow, Poland; 3 Department of Coronary Disease and Heart Failure, Institute of Cardiology, Jagiellonian University Medical College, John Paul II Hospital, 31-202 Krakow, Poland; agackowski@gmail.com; 4 Noninvasive Cardiovascular Laboratory, John Paul II Hospital, 31-202 Krakow, Poland; 5 Department of Cardiac and Vascular Diseases, Institute of Cardiology, Jagiellonian University Medical College, John Paul II Hospital, 31-202 Krakow, Poland

**Keywords:** cardiovascular risk factors, heart failure, major cardiac and cerebral ischemic events, degenerative aortic stenosis, risk stratification, vascular resistance

## Abstract

Background: Cardiovascular disease is a leading cause of heart failure (HF) and major adverse cardiac and cerebral events (MACCE). Objective: To evaluate impact of vascular resistance on HF and MACCE incidence in subjects with cardiovascular risk factors (CRF) and degenerative aortic valve stenosis (DAS). Methods: From January 2016 to December 2018, in 404 patients with cardiovascular disease, including 267 patients with moderate-to-severe DAS and 137 patients with CRF, mean values of resistive index (RI) and pulsatile index (PI) were obtained from carotid and vertebral arteries. Patients were followed-up for 2.5 years, for primary outcome of HF and MACCE episodes. Results: RI and PI values in patients with DAS compared to CRF were significantly higher, with optimal cut-offs discriminating arterial resistance of ≥0.7 for RI (sensitivity: 80.5%, specificity: 78.8%) and ≥1.3 for PI (sensitivity: 81.3%, specificity: 79.6%). Age, female gender, diabetes, and DAS were all independently associated with increased resistance. During the follow-up period, 68 (16.8%) episodes of HF-MACCE occurred. High RI (odds ratio 1.25, 95% CI 1.13–1.37) and PI (odds ratio 1.21, 95% CI 1.10–1.34) were associated with risk of HF-MACCE. Conclusions: An accurate assessment of vascular resistance may be used for HF-MACCE risk stratification in patients with DAS.

## 1. Introduction

With ageing, a reduction in the elastin content and an increase in the collagen content lead to increased arterial stiffness and elevated central as well as peripheral arterial blood pressure [1]. Similarly, chronic low-grade inflammation or metabolic disorders, e.g., glycation of vessel wall proteins, contribute to the stiffening process of large arteries [2,3,4]. Arterial stiffness is a well-known predictor of all-cause mortality, including cardiovascular mortality [5].

Degenerative aortic valve stenosis (DAS) is another condition in which prevalence increases with age [6]. DAS progression, similar to arterial stiffening, is accelerated by common cardiovascular risk factors (CRF) and ageing [6,7,8].

Ultrasonography can easily and non-invasively provide information on vascular resistance indices (resistive index; RI and pulsatile index; PI), that are surrogate markers of arterial stiffness. Peripheral flow parameters can be particularly important in patients with DAS, in whom severely reduced left-ventricle outflow has an impact on the altered vascular system flow pattern [9].

Although both chronological and vascular ageing processes progress in time, they are often not parallel [10]. In patients with CRF, cardiac, and/or arterial disease, vascular ageing outruns the normal ageing process [1,10].

Patients with increased arterial stiffness tend to develop cardiovascular events at a younger age and with a higher mortality rate [1]. However, there are scarce data available as to whether DAS relates to vascular resistance at a higher extent as compared to the ageing process and CRF, and more importantly, whether vascular resistance can contribute to heart failure (HF) episodes and major adverse cardiac and cerebral events (MACCE) in patients with DAS.

We aimed to evaluate impact of vascular resistance on HF and MACCE events in subjects with cardiovascular risk factors and degenerative aortic valve stenosis.

## 2. Materials and Methods

### 2.1. Study Population and Cardiovascular Risk Factors

**Figure 1:**
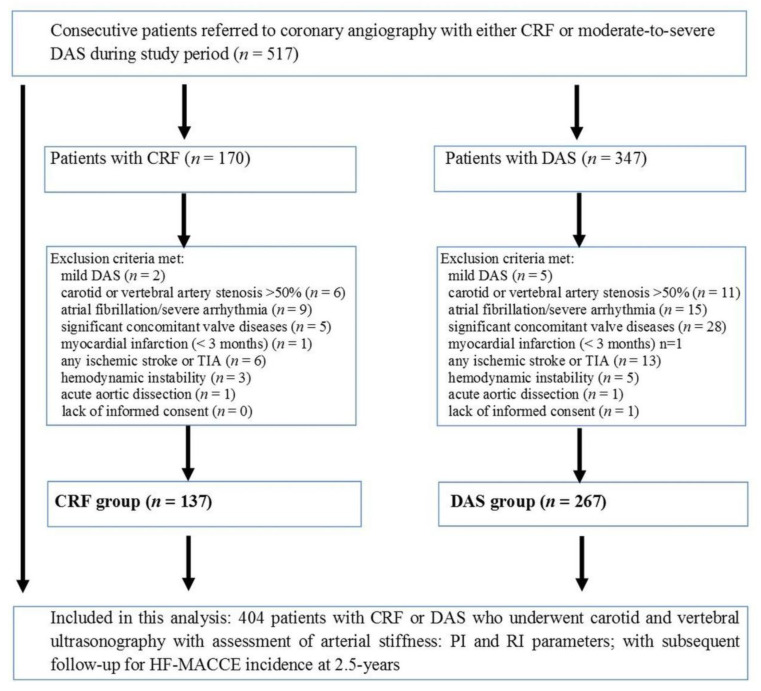
Study flowchart. CRF, cardiovascular risk factors; DAS, degenerative aortic valve stenosis; HF, heart failure; MACCE, major adverse cardiac and cerebral events; PI, pulsatile index; RI, resistive index.

In this single-center prospective study, from January 2016 to December 2018, 517 consecutive patients with either CRF or DAS were assessed. A flowchart of this study is presented in Figure 1.

CRF group was enrolled from patients with suspected or known stable coronary artery disease, with preserved LVEF ≥ 50% admitted to our department for coronary angiography.

Subjects with DAS were eligible if (1) aortic valve area was less than 1.5 cm^2^; they (2) had left ventricular ejection fraction (LVEF) ≥ 50%; and (3) underwent coronary angiography.

The exclusion criteria for both study and control groups included: significant stenosis of any carotid or vertebral artery (exceeding 50% lumen reduction), persistent atrial fibrillation or other severe arrhythmia, significant concomitant valve diseases, ongoing or recent myocardial infarction (<3 months), ischemic stroke or TIA, hemodynamic instability: NYHA class IV or acute heart failure, LVEF < 50%, aortic dissection, and lack of informed consent.

Finally, in 404 study patients, including 267 patients with moderate-to-severe DAS and 137 patients with CRF, distribution of RI and PI registered at carotid and vertebral arteries was evaluated. Patients were followed-up for mean 2.5 years, with primary outcome of HF and MACCE episodes.

The prevalence of CRF, including age, gender, hypertension, diabetes and dyslipidemia, and coronary artery disease was evaluated in both groups. Cardiovascular risk factors were defined as: hypertension (treated, or newly recognized, based on average on three measurements; SBP ≥ 140 mm Hg and/or DBP ≥ 90 mm Hg), diabetes mellitus (treated or newly recognized > 11 mmol/l (200 mg/d) in oral glucose tolerance test, hyperlipidemia (treated or newly recognized—total cholesterol > 4.9 mmol/L (190 mg/dL) and/or LDL > 3.0 mmol/L (115 mg/dL) and/or HDL men < 1.0 mmol/L (40 mg/dL), HDL women < 1.2 mmol/L (46 mg/dL) and/or triglycerides > 1.7 mmol/L (150 mg/dL) [11]. Coronary artery disease was defined as presence on coronary angiography of at least one main coronary artery lumen reduction exceeding 50%.

The study protocol was consistent with the requirements of the Helsinki Declaration, and approved by the local Institutional Ethics Committee. All subjects gave informed consent for participation in the study.

### 2.2. Echocardiography, Carotid and Vertebral Artery Ultrasonography

All patients underwent a complete echocardiographic study in compliance with the guidelines of the European Association of Cardiovascular Imaging [12]. Peak and the mean gradient across aortic valve, aortic valve area (AVA), LVEF were assessed in all subjects.

High-resolution B-Mode, color Doppler, and pulse Doppler ultrasonography of both carotid and vertebral arteries were performed with an ultrasound machine (TOSHIBA APLIO 450) equipped with a linear-array 5–10 MHz transducer in a patient lying in supine position with head tilted slightly backward. Exam was performed by two experienced sonographers who were blinded to subjects’ clinical, echocardiographic, and angiographic characteristics.

Vascular resistance parameters were expressed as averaged RI and PI values calculated bilaterally from internal carotid and vertebral arteries. For this purpose, the peak-systolic (PSV) and the end-diastolic velocities (EDV), as well as vessel diameters were measured within 1.0 to 1.5 cm proximal segment of the internal carotid artery (ICA), and proximal V2 segment of vertebral artery, with a calculation of the pulsatile (PI) and resistive (RI) indexes in each evaluated segment, according to the following equations:

Resistive Index (RI) = PSV – EDV/PSV

Pulsatile Index (PI) = PSV – EDV/[(PSV + 2 × EDV)/3]

The averaged value of RI and PI from four arterial segments was taken into further statistical analysis.

### 2.3. Outcome Data, Follow-Up, and Adverse Cardiovascular Events

During mean observation period of 2.5 years, the incidences of HF episodes and MACCE were recorded.

MACCE was defined as fatal or non-fatal ischemic stroke, myocardial infarction, acute heart failure episode, or cardiovascular death (i.e., any sudden or unexpected death unless proven as non-cardiovascular on autopsy). HF episode was defined as new-onset acute HF incidence or any exacerbation of chronic heart failure requiring in-hospital stay and administration of intravenous medications such as diuretics, dopamine, adrenaline, or dobutamine.

Final visit was conducted through the telephone contact with a patient or pointed family member. For patients lost to follow-up (*n* = 4), the data on patient vital status were obtained from the national health registry.

### 2.4. Statistical Analysis

Data are presented as mean ± standard deviation for continuous variables and as proportions for categorical variables. Differences between mean values were verified using the T-Student, analysis of variance (ANOVA) test, and frequencies were compared by the chi-2 test for independence, as appropriate. The normal distribution of studied variables was determined by the Shapiro–Wilk test. The Spearman’s rank-order correlation was performed for correlation between RI and PI. A receiver-operating characteristic (ROC) analysis was performed to determine the optimal cut-off values (common point of the most distant y = x line with ROC curve) for vascular resistance as potentially associated with DAS. The area under the curve (AUC), cut-offs sensitivity and specificity were calculated. The C statistic with comparison of AUCs was performed for evaluation of models with and without DAS to assess the probability of obtaining arterial stiffness parameters above thresholds. The analysis of risk factors associated with increased PI and RI values was performed with univariate regression analysis. We included age, gender, diabetes mellitus, hypertension, hyperlipidemia, coronary artery disease, LVEF, and DAS as factors potentially associated with vascular resistance. After identification of parameters potentially associated with increased vascular resistance, the multivariable logistic backward regression analysis was used to calculate odds ratio (OR) and 95% confidence interval (95% CI). We used Z-scores to standardize the raw values of age to a normal distribution. We also assessed incidence of HF-MACCE events in groups classified by high versus low PI and RI using univariate logistic regression analysis, followed by the multivariable regression models, with the PI ≥ 1.3 and the RI ≥ 0.7 as referent in all study participants. A 2-sided value of *p* < 0.05 was considered statistically significant. Statistical analyses were performed with Statistica version 13.3 software (TIBCO Software, Palo Alto, CA, USA) and with R Studio 3.6.3 [13].

## 3. Results

### 3.1. Patient Characteristics

The baseline characteristics of the patients are summarized in Table 1. Patients with DAS in comparison to patients with CRFs were significantly older (74.5 vs. 70.0 years, *p* = 0.001) and more often had hyperlipidemia (95.9 vs. 79.6%, *p* < 0.001), while history of myocardial infarction was more frequent in CRF group (31.4 vs. 20.2%, *p* = 0.002). Gender distribution, prevalence of hypertension, diabetes mellitus, and significant coronary artery disease did not differ between the CRF and DAS groups. On echocardiography, baseline LVEF was similar in both study groups, while peak and systolic aortic gradients were significantly higher in DAS vs. CRF groups.

### 3.2. Study Groups and Arterial Stiffness Findings

The RI values were significantly positively correlated with the PI values (r = 0.99, *p* < 0.001).

The RI and PI values in both carotid and vertebral arteries differed significantly between patients with DAS vs. CRF (Table 1). Moreover, mean values of the RI (0.73 ± 0.06 vs. 0.64 ± 0.05, *p* < 0.001) and the PI (1.45 ± 0.23 vs. 1.14 ± 0.16, *p* < 0.001) were significantly higher in patients with DAS, compared to CRF (Table 1).

In line, in patients with moderate (*n* = 32) vs. severe (*n* = 235) DAS, mean values of RI (0.70 ± 0.06 vs. 0.74 ± 0.06; *p* = 0.001) and PI (1.34 ± 0.21 vs. 1.47 ± 0.23; *p* = 0.002) differed significantly.

The optimal cut-off values obtained from ROC analysis best discriminating vascular resistance in CRF vs. DAS patients were RI of 0.7 or higher (sensitivity of 80.5%, specificity of 78.8%) and PI value of 1.3 or higher (sensitivity of 81.3%, specificity of 79.6%).

Univariate regression backward analysis, followed by the multivariate regression analysis, showed associations with the RI ≥ 0.7 and the PI ≥ 1.3 for age, female gender, diabetes, and DAS (Table 2). There was also association between increased arterial stiffness and hyperlipidemia and hypertension in univariate analysis (Table 2).

In multivariable logistic regression backward analysis, DAS confirmed its independent association with high RI and high PI in the multivariate analysis, both in unadjusted and Z-score age-adjusted analysis (Table 2).

**Figure 2:**
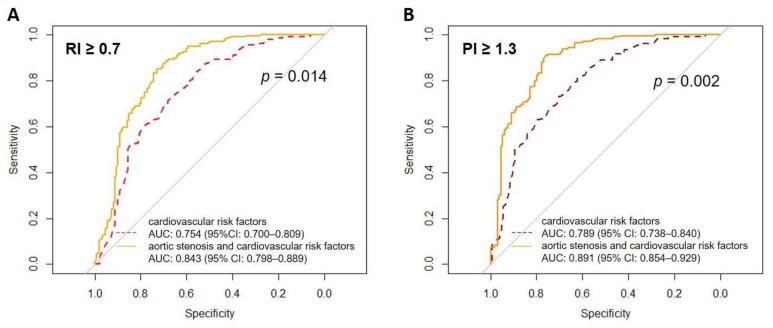
Comparison of area under the curve (AUC) for multivariate models to detect increased Resistive Index (panel (**A**)) and Pulsatile Index (panel (**B**)). Baseline models AUCs for CRF are presented as dashed lines, AUCs for degenerative aortic stenosis are presented as solid lines. Abbreviations: AUC—area under the curve; PI—Pulsatile Index; RI—Resistive Index.

Adding DAS to the model with CRF resulted in a higher predicted probability of the RI ≥ 0.7 (AUC: 0.843 vs. 0.754, *p* = 0.014) and the PI ≥ 1.3 (AUC: 0.891 vs. 0.789; *p* = 0.002) (Figure 2A,B).

### 3.3. Vascular Resistance Properties and the Outcomes

During follow-up period, 68 (16.8%) episodes of HF-MACCE occurred, including 16 (11.7%) in CRF group and 52 (19.5%) in DAS group, *p* = 0.047.

HF-MACCEs were observed in 9 out of 172 patients with RI values below 0.7, as compared to 59 of 232 with RI ≥ 0.7 (5.2% vs. 25.4%, *p* < 0.001), whereas in 9 of 161 patients with PI < 1.3 vs. 59 of 245 patients with PI ≥ 1.3 (5.6% vs. 24.1%, *p* < 0.001).

In both study groups, patients who had HF-MACCE were older, and had higher prevalence of high RI and PI values.

Age, female gender, diabetes, hypertension, DAS, RI ≥ 0.7, and PI ≥ 1.3 showed association with HF-MACCE in univariable analysis (Table 3). In multivariable analysis, high RI (OR, 1.25; 95% CI 1.13–1.37) and PI (OR, 1.21; 95% CI 1.10–1.34), similar to age were independently associated with risk of HF-MACCE.

## 4. Discussion

In the present study, in a subset of patients with moderate-to-severe DAS, similar to patients with CRF, we showed associations between increased carotid arterial resistance, defined as the PI ≥ 1.3 and the RI ≥ 0.7 with heart failure exacerbation episodes and adverse cardiovascular events in mid-term observational period. Therefore, high carotid PI and RI values could be used as surrogate markers of poor cardiovascular prognosis in patients with advanced DAS. The advantage of our concept is that carotid stiffness parameters are easily obtainable non-invasively and are reproducible [14].

Furthermore, ultrasonographic assessment of vascular resistance was also used in former studies, in the setting of large arteries disease or renovascular disease, as the prognostic marker of the outcome [15,16]. Lately, arterial stiffness was also used for risk assessment in patients with COVID infection [17]. 

Our study demonstrated that about three quarters of patients with DAS and cardiovascular risk factors had high RI and PI values, compared to ~ 20% of patients with CRF only. This high distribution of increased arterial stiffness parameters in DAS, as compared to CRF patients, corresponds to ~21–25% relative risk increase of HF-MACCE in mean 2.5-years follow-up period, compared to patients with lower PI and RI values.

We found that moderate-to-severe DAS independently relates to higher vascular resistance, likewise age, female gender, and diabetes. In a study by Yan et al., hypertension (HR 1.71; 95% CI: 1.66–1.76), diabetes (HR: 1.49; 95% CI: 1.44–1.54), and dyslipidemia (HR: 1.17; 95% CI: 1.14–1.21) were all significantly associated with increased risk of developing severe DAS [6]. There was a positive relationship between the severity, number and duration of cardiac risk factors, and risk of DAS [6]. Moreover, gender plays an important role in DAS pathogenesis, development and progression of valvular calcification processes, fibrosis, and hemodynamic severity, left ventricle hypertrophy, and cardiovascular outcomes in men and women [18,19]. Patients with DAS are often older and have more cardiovascular risk factors, systemic hypertension and atherosclerosis, which all show association with increased aortic stiffness and cognitive decline [20,21,22,23].

In contrast to overwhelming studies in favor of an independent role for arterial stiffness in predicting cardiovascular events in healthy elderly and diseased hypertensive, diabetic, or end stage renal disease subjects [24,25], studies concerning potential application of vascular resistance parameters in patients with advanced DAS are innumerous [26,27].

In one research study enrolling 103 asymptomatic patients with moderate-to-severe DAS, arterial stiffness assessed with femoral-carotid pulse wave velocity (PWV) method, showed significantly lower event-free survival in patients with PWV ≥10 m/s compared to those with lower PWV [26]. In line, the Simvastatin and Ezetimibe in Aortic Stenosis study during median 4.3 years observation period demonstrated a higher cardiovascular morbidity rate (hazard ratio 2.13; 95% CI 1.34–3.40) in patients with initially mild-to-moderate DAS and echocardiographically established low systemic arterial compliance [27].

There is some uncertainty about sequence of developing aortic valve calcifications, exposure to atherosclerosis risk factors, and arterial stiffening [1,28]. More recent data demonstrated that increasing arterial stiffness initiates systemic hypertension; thus, it is a cause, not an effect. However, once induced, hypertension leads to further arterial stiffening [28]. In addition, arterial stiffening reflects the vascular ageing process, and the latter one is at least as important as chronological age in cardiovascular events and mortality prediction [29,30]. 

In fact, the results of our study demonstrate that both chronological and vascular age were the only independent risk predictors of HF-MACCE in multivariate analysis.

It is important to realize that DAS is not only a disease of the valve (or heart), but this is a disease of the whole vascular system, and the latter contributes to adverse events. A decrease in elasticity is associated with a multitude of complications, including increased stress on the left ventricle, a gradual increase in blood pressure and, eventually, even end-organ damage through the transmission of harmful pulsation into the microcirculation. Thus, the increase in pulsatility associated with loss of elastic recoil in large blood vessels has detrimental effects on global cardiovascular health [9].

Biological vascular age should be used to select individuals for early prevention of cardiovascular complications with intensification of pharmacotherapy and earlier intervention on the valve [30,31,32]. 

Hypothetically, the risk prediction models may help clinicians develop personalized treatments, i.e., in patients with PI ≥ 1.3 or RI ≥ 0.7, early intervention on DAS could be considered. The treatment of choice could perhaps be transcatheter aortic valve implantation in such a subset of patients, as surgical aortic valve replacement may lead to further significant increase in PWV [31,32].

## 5. Conclusions

Patients with DAS have greater vascular resistance compared to controls with cardiovascular risk factors. Moreover, RI ≥ 0.7 and PI ≥ 1.3 may be used for cardiovascular risk stratification.

## 6. Study Limitations

Our study has obvious limitations, as it consisted of a single-center observational design. Secondly, in general, DAS patients are elderly, which caused difficulties when matching with a control group. For this reason, age-adjustment in multivariate analyses was performed.

## Figures and Tables

**Table 1:** Baseline groups characteristics.

	DAS Group *N* = 267	CRF Group *N* = 137	*p*-Value
**Demographic data**			
Age (years) ± SD	74.5 (8.8)	70.0 (11)	0.001
Female, n (%)	172 (64.4)	87 (63.5)	0.305
Hypertension, n (%)	239 (89.5)	114 (83.2)	0.157
Diabetes, n (%)	86 (32.7)	41 (29.9)	0.556
Hyperlipidemia (%)	256 (95.9)	109 (79.6)	<0.001
Coronary artery disease (%) Previous myocardial infarction, n (%)	111 (41.6) 54 (20.2)	54 (39.4) 43 (31.4)	0.054 0.002
**Selected echocardiographic data**			
Aortic valve area (cm2) ± SD	0.82 ± 0.28	2.5 ± 0.24	<0.001
Peak aortic gradient (mmHg) ± SD	87.8 ± 29	9.6 ± 4.5	<0.001
Mean aortic gradient (mmHg) ± SD	50.5 ± 18.7	4.3 ± 4.2	<0.001
Left ventricular ejection fraction (%) ± SD	60.1 ± 6.6	60 ± 10	0.263
**Carotid and vertebral ultrasonography**			
Mean Resistive Index ± SD	0.73 ± 0.06	0.64 ± 0.05	<0.001
Mean Pulsatile Index ± SD	1.45 ± 0.23	1.14 ± 0.16	<0.001
**Left internal carotid artery**			
Resistive Index ± SD	0.75 ± 0.07	0.64 ± 0.06	< 0.001
Pulsatile Index ± SD	1.52 ± 0.28	1.12 ± 0.17	< 0.001
**Right internal carotid artery**			
Resistive Index ± SD	0.75 ± 0.07	0.64 ± 0.06	< 0.001
Pulsatile Index ± SD	1.52 ± 0.28	1.14 ± 0.17	< 0.001
**Left Vertebral artery**			
Resistive Index ± SD	0.72 ± 0.07	0.65 ± 0.07	<0.001
Pulsatile Index ± SD	1.40 ± 0.25	1.16 ± 0.21	<0.001
**Right vertebral artery**			
Resistive Index ± SD	0.72 ± 0.06	0.64 ± 0.08	<0.001
Pulsatile Index ± SD	1.38 ± 0.23	1.12 ± 0.23	<0.001

CRF, cardiovascular risk factors; DAS, degenerative aortic stenosis.

**Table 2:** Factors associated with increased arterial resistance.

	Univariate OR (95% CI), *p*-Value	Multivariate OR (95% CI), *p*-Value	Multivariate Age-Adjusted OR (95% CI), *p*-Value
**Predictors of RI ≥ 0.7** Age	1.22 (1.12–1.33), <0.001	1.29 (1.20–1.40), <0.001	1.29 (1.20–1.40), <0.001
Female gender	1.10 (1.01–1.19), 0.025	1.07 (0.99–1.16),0.070	1.07 (0.99–1.16),0.071
Diabetes	1.15 (1.04–1.27), 0.004	1.10 (1.02–1.18), 0.018	1.10 (1.02–1.19), 0.018
Hypertension	1.17 (1.06–1.29), 0.002	1.07 (0.99–1.16), 0.076	1.07 (0.99–1.16), 0.076
Hyperlipidemia	1.27 (1.15–1.39), <0.001	1.06 (0.98–1.55), 0.155	1.06 (0.98–1.15), 0.155
Coronary artery disease	1.07 (0.97–1.18), 0.173	-	
Previous myocardial infarction	1.00 (0.91–1.10), 0.944	-	
Left ventricular ejection fraction	1.05 (0.95–1.16), 0.319	-	
Aortic valve stenosis	2.49 (1.64–3.78), <0.001	1.65 (1.53–1.79), <0.001	1.66 (1.53–1.79), <0.001
**Predictors of PI ≥ 1.3**			
Age	1.44 (1.32–1.58), <0.001	1.25 (1.16–1.35), <0.001	1.25 (1.16–1.35), <0.001
Female gender	1.16 (1.05–1.28), 0.002	1.12 (1.04–1.20), 0.004	1.12 (1.04–1.21), 0.004
Diabetes	1.17 (1.06–1.29), 0.001	1.11 (1.03–1.19), 0.009	1.11 (1.03–1.19), 0.009
Hypertension	1.18 (1.07–1.29), 0.001	1.06 (0.98–1.15), 0.117	1.06 (0.98–1.15), 0.118
Hyperlipidemia	1.29 (1.18–1.42), <0.001	1.09 (1.01–1.18), 0.027	1.09 (1.01–1.18), 0.027
Coronary artery disease	1.10 (1.00–1.21), 0.059	-	
Previous myocardial infarction	1.01 (0.92–1.11), 0.934	-	
Left ventricular ejection fraction	1.04 (0.95–1.15), 0.388	-	
Aortic valve stenosis	1.79 (1.65–1.94), <0.001	1.67 (1.54–1.80), <0.001	1.67 (1.54–1.80), <0.001

**Table 3:** Univariate and multivariate logistic regression analysis of factors associated with heart failure episodes (HF) and major adverse cardiac and cerebral events (MACCE).

Parameter	Univariate Analysis	Multivariate Analysis with RI	Multivariate Analysis with PI
	OR (95% CI), *p*-Value	OR (95% CI), *p*-Value	OR (95% CI), *p*-Value
Age	1.23 (1.12–1.35), 0.001	1.13 (1.03–1.25), 0.014	1.16 (1.04–1.26), 0.008
Female gender	1.12 (1.02–1.23), 0.019	1.07 (0.98–1.18), 0.133	1.07 (0.97–1.18), 0.163
Diabetes	1.11 (1.01–1.23), 0.029	1.05 (0.96–1.16), 0.293	1.06 (0.96–1.16), 0.269
Hypertension	1.12 (1.02–1.23), 0.025	1.07 (0.97–1.17), 0.169	1.07 (0.97–1.18) 0.162
Hyperlipidemia	1.01 (0.92–1.12), 0.783	-	-
Coronary artery disease	1.07 (0.97–1.18), 0.172	-	-
Previous myocardial infarction	1.01 (0.91–1.11), 0.919	-	-
Left ventricle ejection fraction	1.04 (0.95–1.15), 0.384	-	-
Aortic valve stenosis	1.10 (1.00–1.21), 0.047	1.07 (0.95–1.19), 0.253	1.05 (0.94–1.18), 0.407
Peak aortic gradient	1.10 (0.98–1.24), 0.106	-	-
RI ≥ 0.7	1.30 (1.19–1.43),0.001	1.25 (1.13–1.37), <0.001	-
PI ≥ 1.3	1.28 (1.16–1.40), <0.001	-	1.21 (1.10–1.34), <0.001

## Data Availability

The data presented in this study are available on request from the corresponding author. The data are not publicly available due to privacy.
